# Supplementary material for: Dietary Practices and Adolescent Obesity in Secondary School Learners at Disadvantaged Schools in South Africa: Urban–Rural and Gender Differences
Source: Int J Environ Res Public Health. 2020 Aug 13;17(16):5864. doi: 10.3390/ijerph17165864 (PMC7460370; doi:10.3390/ijerph17165864)
Supplement: Supplementary file 1 [file ijerph-17-05864-s001.docx]

Supplementary Table 1. Dietary practices associated with body weight status of secondary school adolescents in the Eastern Cape

| Factors | Body weight status | | | | | | | Bivariate logistic regression | | |
| --- | --- | --- | --- | --- | --- | --- | --- | --- | --- | --- |
|  | Underweight  n (%) | Normal weight  n (%) | Overweight  n (%) | Obese  n (%) | Rao-Scott Chi-square p-value | Overweight/ Obese  n (%) | Rao-Scott Chi-square p-value | Risk of underweight  OR (95% CI)  n=323 | Risk of overweight / obese  OR (95% CI) | Risk of obese  OR (95% CI) |
| Usual weekly consumption of breakfast | | |  |  |  |  |  |  |  |  |
| Never | 12 (9.3) | 74 (57.4) | 28 (21.7) | 15 (11.6) | 0.014* | 43 (33.3) | 0.003** | Ref | Ref | Ref |
| 1-2 days | 13 (4.9) | 168 (63.9) | 49 (18.6) | 33 (12.5) |  | 82 (31.2) |  | 0.59 (0.25-1.42) | 1.12 (0.69-1.84) | 1.29 (0.60-2.77) |
| 3-4 days | 21 (11.9) | 121 (68.8) | 23 (13.1) | 11 (6.3) |  | 34 (19.3) |  | 1.53 (0.55-4.30) | 0.60 (0.31-1.18) | 0.60 (0.21-1.73) |
| 5 days | 58 (8.8) | 437 (66.6) | 115 (17.5) | 46 (7.0) |  | 161 (24.5) |  | 1.10 (0.46-2.64) | 0.82 (0.49-1.36) | 0.68 (0.34-1.37) |
| Learners usual eating practices during the weekdays | | | |  |  |  |  |  |  |  |
| 3 Meals a day | 63 (8.8) | 470 (65.4) | 123 (17.1) | 63 (8.8) | 0.785 | 186 (25.9) | 0.677 | Ref | Ref | Ref |
| 2 Meals a day | 25 (7.8) | 208 (64.6) | 59 (18.3) | 30 (9.3) |  | 89 (27.6) |  | 0.95 (0.57-1.58) | 1.15 (0.81-1.63) | 1.10 (0.69-1.75) |
| 1 Meal a day | 17 (10.2) | 108 (65.1) | 31 (18.7) | 10 (6.0) |  | 41 (24.7) |  | 1.23 (0.76-2.00) | 0.94 (0.72-1.22) | 0.66 (0.36-1.22) |
| Learners usual eating practice during the weekend | | | |  |  |  |  |  |  |  |
| 3 Meals a day | 61 (8.1) | 496 (65.7) | 128 (17.0) | 70 (9.3) | 0.573 | 198 (26.2) | 0.244 | Ref | Ref | Ref |
| 2 Meals a day | 31 (9.8) | 197 (62.1) | 61 (19.2) | 28 (8.8) |  | 89 (28.1) |  | 1.29 (0.80-2.10) | 1.14 (0.92-1.41) | 1.00 (0.59-1.68) |
| 1 Meal a day | 13 (8.8) | 101 (68.7) | 25 (17.0) | 8 (5.4) |  | 33 (22.4) |  | 1.11 (0.73-1.69) | 0.81 (0.54-1.21) | 0.57 (0.21-1.55) |
| Number of times snacks are usually eaten in a day | | | |  |  |  |  |  |  |  |
| None | 31 (6.9) | 305 (67.8) | 71 (15.8) | 43 (9.6) | 0.698 | 114 (25.3) | 0.968 | Ref | Ref | Ref |
| Once a day | 33 (9.8) | 213 (63.4) | 62 (18.5) | 28 (8.3) |  | 90 (26.8) |  | 1.51 (0.79-2.87) | 1.11 (0.73-1.69) | 0.89 (0.44-1.80) |
| 2 | 34 (9.2) | 238 (64.3) | 66 (17.8) | 32 (8.6) |  | 98 (26.5) |  | 1.40 (0.76-2.58) | 1.10 (0.74-1.62) | 0.92 (0.50-1.69) |
| 3+ | 7 (9.1) | 49 (63.6) | 18 (23.4) | 3 (3.9) |  | 21 (27.3) |  | 1.32 (0.39-4.45) | 1.07 (0.66-1.74) | 0.38 (0.11-1.34) |
| Number of takeaways in past week |  |  |  |  |  |  |  |  |  |  |
| None | 21 (8.0) | 171 (65.5) | 45 (17.2) | 24 (9.2) | 0.374 | 69 (26.4) | 0.863 | Ref | Ref | Ref |
| 1-2 | 28 (7.6) | 242 (65.8) | 59 (16.0) | 39 (10.6) |  | 98 (26.6) |  | 1.00 (0.53-1.87) | 1.16 (0.80-1.68) | 1.26 (0.81-1.97) |
| 3-4 | 23 (8.0) | 194 (67.6) | 53 (18.5) | 17 (5.9) |  | 70 (24.4) |  | 1.01 (0.69-1.47) | 0.98 (0.61-1.56) | 0.64 (0.33-1.26) |
| 5+ | 30 (10.2) | 184 (62.6) | 57 (19.4) | 23 (7.8) |  | 80 (27.2) |  | 1.31 (0.80-2.13) | 1.12 (0.85-1.47) | 0.86 (0.48-1.55) |

* Statistically significant at p < 0.05;  **Statistically significant at p < 0.01;***p<0.001; n=number of individual characteristics
